# Supplementary material for: Novel Polyclonal Antibody Raised against Tetrodotoxin Using Its Haptenic Antigen Prepared from 4,9-anhydrotetrodotoxin Reacted with 1,2-Ethaneditiol and Further Reacted with Keyhole Limpet Hemocyanin
Source: Toxins (Basel). 2019 Sep 20;11(10):551. doi: 10.3390/toxins11100551 (PMC6832204; doi:10.3390/toxins11100551)
Supplement: Supplementary file 1 [file toxins-11-00551-s001.pdf]

Supplementary Materials: Novel Polyclonal Antibody Raised against Tetrodotoxin Using Its Haptenic Antigen Prepared from 4,9-anhydrotetrodotoxin Reacted with 1,2-Ethaneditiol and Further Reacted with Keyhole Limpet Hemocyanin

Shigeru Sato, Suzuka Takaishi, Ko Yasumoto and Shugo Watabe

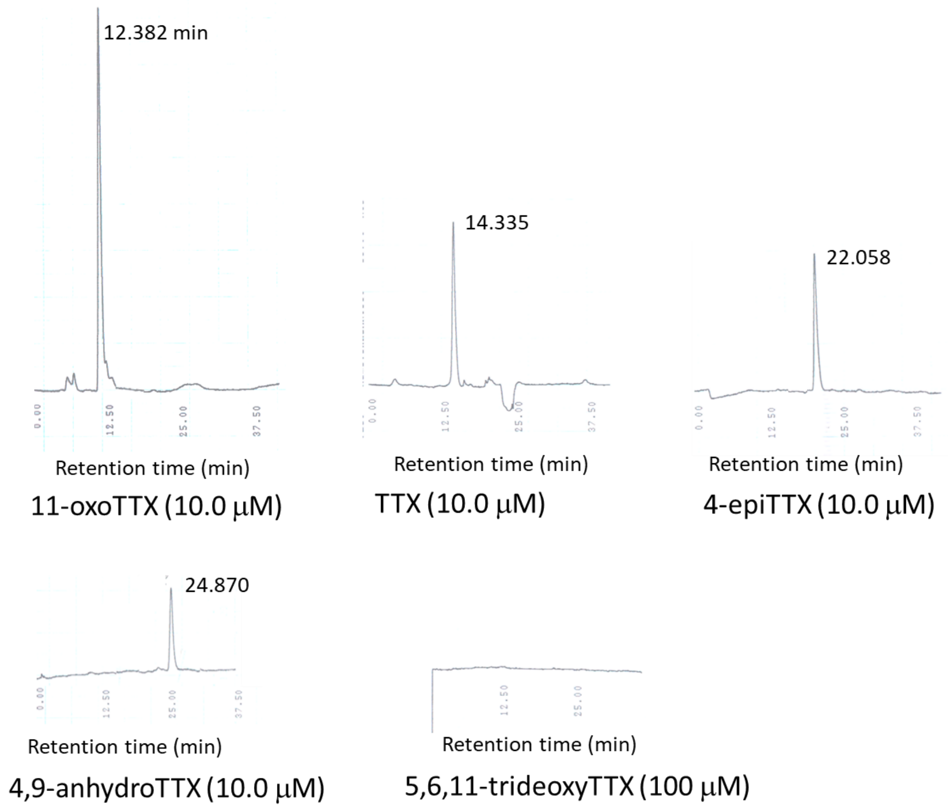

Figure S1. Fluorometric HPLC analysis of isolated TTX analogs. In Figure 5, these isolated toxins were analyzed on ELISA.

Table S1. Reaction of 4,9anhydro-TTX with DTT expressed as concentration of the remaining free toxins measured by HPLC-FLD in function of time.

Min	Conc. (μM)			
	4,9anhydro-TTX	TTX	6epi-TTX	4epi-TTX
0	1099	ND	ND	ND
15	162	ND	ND	ND
30	ND	ND	ND	ND

Reaction mixture was analyzed by the fluorometric HPLC [38]. ND means not detected.

Table S2. The reactivity of the antibody against KLH in ELISA.

	**OD (n = 3)**			**OD (n = 3)**	
TTX (nM)	mean	SE	KLH (ppm)	mean	SE
0	0.702	0.023	0	0.702	0.017
1	0.698	0.019	0.1	0.701	0.021
10	0.423	0.023	1	0.67	0.022
100	0.225	0.023	10	0.67	0.021
1000	0.151	0.023	100	0.607	0.019
Bound 0 %	0.124	0.021	Bound 0 %	0.124	0.017

TTX and KLH with several concentrations were separately analyzed on the same ELISA plate. Background OD (absence of biotin-TTX) is expressed as “Bound 0%.
